# Serum in Pulp Cavities

**Published:** 1860-01

**Authors:** 


### SERUM IN PULP CAVITIES.

Since reporting the experiments (published in the October number) demonstrating the permeability of dental tissues; on three different occasions, after thoroughly removing the nerves from teeth, we have washed out the cavities by injecting water through a syringe; dried them perfectly with tissue paper, and then with fine root-pluggers, packed cotton tightly in the pulp chambers, and filled the cavities of decay with wax. Each patient was then dismissed until the following day, when, after wiping the parts about the tooth perfectly dry, napkins were introduced, as if preparing to fill a tooth. The wax and cotton were then removed, and the cavities of decay alone filled carefully with Slayton's preparation. The patients were dismissed again and requested to return in two days. At the expiration of that time the same precautions having been observed with regard to external moisture, the fillings were removed, and on passing a portion of *perfectly white* tissue paper into the pulp cavities, it absorbed a fluid that gave a *rosy tint* to the paper, indicating in the most deciding manner the presence of *serum* there.—*Dental Cosmos.*

Carefully conducted experiments are highly instructive and satisfactory. The one detailed above shows pretty clearly that when a living soft tissue is torn or cut, hemorrhage is likely to follow. It may even take place when a dead part is separated from a living one; and this experiment proves that the dental nerves are not an exception to the rule. It seems also that the serum is discharged from the wound for some time after the flow of red globules ceases. We had inferred as much from experiments less carefully conducted; for example, we noticed that when a cut finger was dressed with a "*perfectly white*" linen rag "it absorbed a fluid that gave a *rosy tint* to it, indicating in the most decided manner the presence of *serum* there."

But the flow of serum will be arrested in process of time; and the arrest may be quickened by the use of appropriate astringents or escharotics. W.

